# The interplay of salt stress and *Azolla* aqueous extract on ionic balance, secondary metabolism, and gene expression in wheat seedlings

**DOI:** 10.1186/s12870-025-06688-3

**Published:** 2025-05-23

**Authors:** Khalil M. Saad-Allah, Sherien E. Sobhy, Elsayed E. Hafez, Thorya A. Fallatah, Abeer M. Kutby, Ghalia S. Aljeddani, Fayza R. ALgthami, Ameina S. ALmoshadak, Wessam F. Felemban, Heba H. Elsehely

**Affiliations:** 1 Botany Department, Faculty of Science, Tanta University, Tanta, 31527 Egypt; 2 Plant Protection and Bimolecular Diagnosis Department, Arid Lands Cultivation Research Institute, City of Scientific Research and Technological Applications, 21934 New Borg El‑Arab, Egypt; 3 Department of Biological Science, College of Science, University of Jeddah, Jeddah, Saudi Arabia; 4 Department of Environmental Sciences, College of Science, University of Jeddah, Jeddah, Kingdom of Saudi Arabia; 5 Department of Biological Sciences, Faculty of Sciences, King Abdulaziz University, Jeddah, Kingdom of Saudi Arabia; 6 Immunology Unit, King Fahd Medical Research Center, King Abdulaziz University, Jeddah, Saudi Arabia

**Keywords:** *Azolla filiculoides* extract, Salinity, Antioxidants, Secondary metabolites, qRT-PCR

## Abstract

**Background:**

The resilience of plants against environmental challenges, particularly salinity and dehydration, is crucial for global food security. This study delves into the intricate interaction between NaCl-induced salinity and *Azolla* aqueous extract (AAE). In a pot trial, wheat kernels were primed with deionized water or 0.1% AAE for 21 h. Seedlings underwent various treatments; tap water, 250 mM NaCl, AAE priming and spray, and combined AAE with NaCl treatments. Seedlings were analyzed for ionic balance, secondary metabolism, antioxidant efficacy, and molecular response to experimental treatments.

**Results:**

GC-MS analysis of AAE revealed key components like γ-aminobutyric acid and benzenedicarboxylic acid. Exposure to 250 mM NaCl significantly reduced N, P, Ca, K, and the K/Na ratio, while increases in Mg and Na. Also, salinity significantly decreased TAC, DPPH activity, and AsA levels while increasing GB in wheat seedlings. Additionally, salinity increased flavonoids, saponins, and anthocyanins but non-significantly decreased phenols. qRT-PCR analysis revealed upregulation of DRF1, CBF3, HQT, CHS, and FLS genes and downregulation of CBF4 and CHI genes by salinity. AAE treatments, alone or combined with salt stress, mitigated Na accumulation (31.50 and 32.87% compared to stressed seedlings), improved N and P levels, alleviated Mg, K/Na, and GB imbalances, and enhanced antioxidant potentials. Combined AAE and NaCl treatments effectually restored antioxidant potentials and regulated secondary metabolites and gene expressions, sustaining enhancement of ionic equilibrium, antioxidant defenses, and molecular responses in salt-stressed wheat.

**Conclusions:**

Overall, AAE can be exploited as a green approach for sustaining normal metabolism and gene expression of wheat seedlings in saline soils.

**Supplementary Information:**

The online version contains supplementary material available at 10.1186/s12870-025-06688-3.

## Background

The agricultural sector on a global scale is encountering numerous obstacles arising from the proliferation of adverse unfavorable environmental conditions including high temperatures, freezing events, extreme cold, excessive salt accumulation, soil sodicity, air and soil pollution, and many other causes. These challenges have become particularly pronounced due to the ongoing climatic changes experienced in the present era. The impact of climate change is intricately interlinked with agricultural production, as it negatively impacts crop productivity, water consumption, biodiversity, and soil health. The overall consequence of climate change on global agriculture is expected to be predominantly adverse, and thus pose a threat to food security [1].

Soil salinity, distinguished by an elevated presence of soluble salts within the soil matrix, represents a substantial worldwide concern that exerts profound effects on both agricultural yield and the long-term viability of ecosystems. Salt-affected soils encompass a vast area of 935 million hectares worldwide [2], with over 70% of this area found in arid and semi-arid regions such as India, Pakistan, Australia, African countries, China, Middle-Eastern countries, and the USA [3]. Moreover, the extent of salinized soils continues to expand at a rate of 1–2 million hectares per year, and this rate is projected to escalate further in the coming decades due to the influence of climate change [4]. The causes of soil salinity can be attributed to natural processes (known as primary salinity), irrigation water quality, agricultural water management practices, fertilizer usage, and the impacts of climate change such as reduced rainfall, elevated temperatures, and rising sea levels [5].

Salt stress is known to adversely affect various physiological processes in plants, including seed germination, growth, photosynthesis, and nutrient and water uptake through vascular systems. When crops are cultivated in saline soils, they encounter osmotic stress, nutritional imbalances, and ion toxicity, all of which can severely reduce crop yields. The severity and nature of these impacts can differ significantly based on the specific crop species, growth stages, and their susceptibility to saline conditions [6]. In response to environmental challenges, plants have developed inherent mechanisms to cope with salinity stress. Key defensive strategies include root-based ion exclusion and the regulation of ion uptake and transport through various proteins, such as carrier proteins, channel proteins, antiporters, and symporters. Additionally, plants exhibit tissue tolerance to elevated ion concentrations [7]. To maintain cellular stability under stress, they produce and accumulate compatible solutes, such as proline, glycine betaine, sugars, and polyols [8].

Adaptation to salinity stress involves a multifaceted approach, with modifications across morphological, physiological, biochemical, and molecular levels. The acquisition of salt tolerance is largely mediated by the management of cytoplasmic ion concentrations. This includes maintaining ion balance, compartmentalization, osmotic regulation, and enhancing antioxidant systems, particularly through increased scavenging of reactive oxygen species [9]. Furthermore, the response to salinity stress is regulated by a variety of intrinsic phytohormones, including abscisic acid, auxin, salicylic acid, jasmonic acid, cytokinins, gibberellins, ethylene, and brassinosteroids, which play crucial roles in modulating plant responses and promoting resilience to saline environments [10]. Exposure to such stress also leads to the upregulation of specific genes and transcription factors associated with salinity adaptation, enabling plants to acclimate effectively to saline conditions [8].

The increasing global population necessitates sustainable food production, driving extensive research into soil salinity and effective mitigation strategies in agriculture [11]. To address the challenges posed by saline soils, various innovative approaches have emerged, including the use of biochar, nanoparticles, endophytic microorganisms, salt chelators, hydrogel composites, organic amendments, and crop residues [12–15], in addition to many other approaches. The implementation of these mitigation strategies has been shown to have positive effects on crop growth and yield. These strategies achieve this by activating germination enzymes, balancing ROS levels, promoting the manufacture of compatible solutes, boosting antioxidant defense systems, and promoting the production of aquaporins in seeds and root cells, contributing to efficient water absorption, particularly under challenging saline conditions [16, 17]. Among these, the selection of *Azolla* aqueous extract (AAE) as a treatment is particularly promising due to its natural properties that enhance plant resilience and nutrient availability in saline conditions.

*Azolla* is a floating aquatic fern found in both temperate and tropical regions, characterized by its unique dorsal leaves that contain cavities housing cyanobacteria capable of fixing atmospheric nitrogen through biological processes [18]. Due to its nitrogen-fixing abilities, *Azolla* species are widely utilized as efficient green manure and biofertilizers in flooded agricultural systems, particularly in rice cultivation [19]. Notably, Gaind and Singh [20] have highlighted the beneficial effects of *Azolla* in a rotating rice-wheat cropping system, demonstrating its positive impact on wheat cultivation. In addition to its role in nitrogen fixation, *Azolla* provides essential nutrients, including vitamins, growth stimulants, essential amino acids, and various mineral ions such as (Ca, Mg, K, P, Fe, and Cu [21]. While existing research has documented the role of *Azolla filiculoides* in the phytoremediation of metal-contaminated soils and its ability to enhance crop yield under drought conditions, a comprehensive review of the literature reveals a lack of studies investigating its potential to mitigate the effects of salinity on growth and development across various crop species.

Bread wheat (*Triticum aestivum* L.) is a vital global food crop, extensively cultivated and consumed worldwide. As the second most cultivated cereal after maize, wheat serves as a staple food for a significant portion of the global population, contributing approximately 20% of daily caloric and protein intake. Its cultivation occurs on both non-saline and saline soils, covering about 214.79 million hectares [22]. Among field crops, wheat is particularly sensitive to salinity, which adversely impacts growth and development, resulting in reduced productivity or even total crop failure under severe salinity conditions [23]. Despite the inherent genetic diversity for salt tolerance within the wheat genome, modern elite cultivars often struggle in high salinity environments, with many genotypes yielding minimal grain production when exposed to salinity levels exceeding 150 mM NaCl [24].

Various strategies have been implemented to enhance wheat growth and yield by targeting physiological and biochemical processes, such as vacuolar Na⁺ sequestration, Na⁺ exclusion, K⁺ retention, osmoregulation, and improved photosynthetic efficiency [25]. However, a promising approach involves utilizing natural plant extracts as amendments to alleviate the detrimental effects of salt stress on wheat growth and production. This strategy not only offers an economical solution but also effectively promotes desirable characteristics in wheat. Consequently, the aim of this study is to evaluate the effectiveness of *Azolla filiculoides* aqueous extract (AAE) when applied as both a priming treatment and foliar application in mitigating the impact of salt stress on ionic equilibrium, antioxidant capacity, osmoregulatory molecules, specific secondary metabolites, and the expression of some salt-responsive genes in wheat subjected to elevated salinity levels (250 mM NaCl). Furthermore, this research seeks to analyze the phytochemical content of AAE and assess its capacity to mitigate salt-induced toxicity in wheat plants, elucidating the mechanisms through which these phytochemicals confer salt tolerance.

## Methods

### Azolla extract preparation

Following the collection of *Azolla filiculoides* from an irrigation canal in Tanta City, Gharbia, Egypt, the plants underwent a meticulous cleaning process. They were thoroughly washed to remove any impurities and then dried in a shady place for 3 days, then in an air-forced oven for 4 days. The dried plants were subsequently pulverized into particles of 2 mm in diameter.

To prepare the *A. filiculoides* extract, we utilized a solvent mixture of ethanol and water in a volumetric ratio of 97.5:2.5, based on the methodology described in our previous publication [8]. The resulting solution was filtered to remove any solid residues. Following filtration, the solution was dehydrated under reduced pressure using a rotary evaporator set at a temperature of 40 °C. The dehydrated extract was then redissolved in distilled water to create a stock solution of aqueous *Azolla* extract (AAE) at a concentration of 0.1%.

### GC-MS analysis of AAE phytochemical constituents

The phytochemical composition of AAE was identified by following the approach described by Nessem et al. [26]. A Clarus 580/560S gas chromatograph/mass spectrometer (PerkinElmer, Inc. Waltham, MA, USA) was utilized for this purpose. The phytochemical components of the extract were separated using an Elite-5MS column (30 m × 0.25 mm × 0.25 μm film thickness). The temperature of the oven was initially set at 80 °C and held for 7 min., followed by an increase of 10 °C/min. until 140 °C withhold for 1 min. Subsequently, the temperature was raised to 200 °C at a rate of 10 °C/min. withhold for 1 min., and then to 280 °C at a rate of 5 °C/min. withhold for 10 min. The temperature of the input and transfer lines was maintained at 250 °C. Helium at a constant flow rate of 1 ml/min. was used as the carrier gas.

Using the GC in split mode (1:20), the autosampler (AS3000) was used to load 1.0 µl of AAE automatically. Mass spectra under Electron Ionization (EI) were acquired using an ionization energy of 70 eV, spanning m/z 40–650 in full scan mode. The temperature of the ionization chamber was maintained at 200 °C. Retention times and mass spectra of the phytocomponents within AAE were cross-referenced with the WILEY 09, replib, and NIST 11 mass spectral databases to ascertain the identity of each component. The phytochemical components of the AAE are presented in Table 1.

**Table 1:** qRT-PCR specific primers sequence used in this study

Gene name	Abbreviation	Direction	Sequences 5 ـــــ 3
Chalcone synthase	CHS	F	AGGCTAACAGAGGAGGGTA
		R	CCAATTTACCGGCTTTCT
Chalcone isomerase	CHI	F	TGGTGGCCTAGACAACGATGAGTT
		R	TCACACTCCCAACTTGGTTTCCCT
Flavonol synthase	FLS	F	TTAAAGGAAGGTCTCGGTGGCGAA
		R	TCATTGGTGACGATGAGTGCGAGT
Dehydration responsive factor 1	DRF1	F	TGGAGCAGAGGAAAGTACCC
		R	CATCTCCCTTGGGGTTTTG
C-repeat binding factor 3	CBF3	F	CGAACGACGCTGCCATGCTC
		R	GGACCCAGACGACGGAGATA
C-repeat binding factor 4	CBF4	F R	CGACGCCAAGGACATTCA
			CTCTCTTCCCTCCTCTCATCTT
Hydroxycinnamoyl-CoA quinate hydroxycinnamoyl transferase	HQT	F	CCCAATGGCTGGAAGATTAGCTA
		R	CATGAATCACTTTCAGCCTCAACAA
Reference gene	ß-Actin	F	GTGGGCCGCTCTAGGCACCAA
		R	CTCTTTGATGTCACGCACGATTTC

### Experimental setup and treatments

The bread wheat kernels (*Triticum aestivum* L. cv Sakha 96) employed in this investigation were sourced from the Agricultural Research Centre, Sakha, Egypt. To confirm sterility, the kernels were drenched in a 0.1% HgCl_2_ solution for 10 min. Following thorough rinsing with deionized water, the sterilized kernels were subjected to priming in deionized water or a 0.1% AAE solution for 21 h. Subsequently, post priming, these kernels were sown into plastic containers measuring 20 × 20 cm, filled with 8 kg clay-sandy soil mixture at a ratio of 2:1. Watering with tap water continued until the seedlings were completely established (6 days).

The pots were then allocated into two main groups: one primed with deionized water and the other primed with AAE as a pretreatment. The water-primed group was further divided into four sub-treatments: tap water, 250 mM NaCl, AAE spray, and AAE spray + NaCl. On the other hand, within the AAE-primed group, two sub-treatments were administered: tap water and 250 mM NaCl. The experiment encompassed a total of six treatments, each replicated three times following a completely randomized design. The irrigation solution utilized for each treatment equated to 70% of the corresponding field capacity.

AAE was administered to the upper leaf surface via a manual atomizer post-sunrise until saturation. This spraying regimen was repeated three times at five-day intervals. Subsequently, after 21 days, the plants were harvested for mineral, biochemical, and molecular analyses.

### Mineral content analysis

These dry powdered leaves of wheat seedlings underwent wet digestion utilizing a mixture of 70% HNO_3_ and 30% H_2_O_2_ (5:3 v/v). Subsequently, the resulting digestates underwent filtration and were then neutralized using 4 N NaOH and phenolphthalein as an indicator. The volume was adjusted to a specific value using distilled water.

The determination of nitrogen (N) levels was conducted via calorimetric analysis employing Rochelle reagent, with NH4Cl employed as the reference standard, while the quantification of phosphorus (P) content was accomplished using molybdenum blue reagent and KH_2_PO_4_ as the standard, following the methodology outlined by Allen et al. [27].

To determine the concentration of calcium (Ca), magnesium (Mg), potassium (K), and sodium (Na) in the digested samples, an inductively coupled plasma-optical spectrophotometer (Polyscan 61 E, Thermo Jarrell-Ash Corp., Franklin, MA, USA) was employed. The elemental content of the samples was expressed in mg g^− 1^ DM. Additionally, the K/Na ratio was calculated based on the measured concentrations of K and Na ions.

### Antioxidative potential of wheat leaves

The antioxidative properties, including total antioxidant capacity and radical scavenging activity, as well as the levels of the antioxidant molecules ascorbic acid and glycine betaine in wheat leaves were assayed. The total antioxidant capacity (TAC) in the ethanolic extracts of the powdered leaf was evaluated using the phosphomolybdate assay [28]. A 300 µl aliquot of the extract was mixed with the phosphomolybdate reagent and incubated at 95 °C for 90 min. After cooling, the absorbance of the mixture was monitored at 765 nm. The TAC was expressed as µg of ascorbic acid equivalents (AAE) g^− 1^ DM.

The radical scavenging activity of the extracts was determined following the method outlined by Sermakkani and Thangapandian [29]. A 0.1 ml aliquot of the extract was mixed with 1,1-diphenyl-2-picrylhydrazyl (DPPH) radical (0.67% in methanol). After incubation in the dark for 1 h, the absorbance was measured at 515 nm and the DPPH activity was then calculated as a percentage (%).

The quantification of ascorbic acid (AsA) content in the wheat leaf powders was carried out using the method developed by Oser [30]. AsA was extracted using a 5% aqueous sulfosalicylic acid solution. The extracts were then allowed to react with Na-molybdate (2%), H_2_SO_4_ (0.15 N), and Na_2_HPO_4_ (1.5 mM). The mixtures were incubated at 60 °C for 40 min., and after centrifugation, the absorbance was measured at 660 nm. The content of AsA was calculated as mg g^− 1^ DM using a calibration curve from AsA.

The determination of glycine betaine (GB) content was conducted following the method described by Grieve and Grattan [31]. Leaf powders were mechanically agitated in deionized water for 24 h, followed by centrifugation. The supernatants were diluted (1:1) with 2 N HCl. A 1 ml aliquot of the extract was mixed with 3 ml of cold KI-I_2_ reagent in an ice bath for 1 h. The mixture was then centrifuged in a cooling centrifuge, and the resulting periodide crystals were dissolved in 9 ml of 1,2-dichloroethane. The absorbance was measured at 365 nm against standard glycine betaine concentrations, and the concentration was calculated as µg g-1 DM.

### Determination of secondary metabolites

The quantification of total flavonoids in wheat leaves was performed using the aluminum chloride technique [32]. The ethanolic extract of the leaves was combined with 95% ethanol, 10% AlCl_3_, and 1000 mM potassium acetate. After 30 min., the absorbance was measured at 415 nm against a blank containing distilled water instead of AlCl_3_. A calibration curve was constructed using quercetin as the standard flavonoid, and the total flavonoid content was determined (mg g^− 1^ DM).

The estimation of total phenolic content in wheat leaves was carried out using the method outlined by Jindal and Singh [33]. The ethanolic extract of the leaves was mixed with Folin–Ciocalteu’s reagent and Na_2_CO_3_ (20%), and the absorbance was measured at 650 nm. Total phenolic content (mg g^− 1^ DM) was determined using a standard curve generated with gallic acid.

To assess the saponin content in the ethanolic extract, it was allowed to reacte with 8% vanillin and 72% sulfuric acid. The resulting mixture was incubated at 60 °C for 10 min., cooled in an ice-cold water bath, and the absorbance was measured at 544 nm. A standard curve by cholesterol was used to quantify the saponin content as mg g^− 1^ DM [34].

Acidic methanol was employed for the extraction of anthocyanin content from wheat fresh leaf tissues. The homogenates were centrifuged and the difference in absorbance at 525 nm and 585 nm was recorded as the variation in anthocyanin content among the investigated samples [35].

### qRT-PCR analysis of stress-related genes

The extraction of total RNA was mediated by the RNeasy Mini Kit (Qiagen) according to the supplier’s guidance. The reverse transcription procedures were used to create complementary DNA (cDNA) from RNA. This was achieved in a total volume of 20 µl employing a thermocycler (MJ Research, Inc., PTC-100™ Programmable thermal controller, USA). The cDNA reaction sequence consisted of an initial enzyme activation cycle at a temperature of 42 °C for 1 h, followed by a subsequent enzyme inactivation cycle at a temperature of 80 °C for 15 min.

The qRT-PCR was conducted in triplicate using the SYBR Green PCR Master Mix from Fermentas, USA. Each reaction contained a 25 µl reaction mixture containing the primer pairs (DRF1, CBF3, CBF4, HQT, CHS, CHI, and FLS) (Table 1). Data were captured during the extension phase. The experiment was conducted using a Rotor-Gene 6000 machine (QIAGEN, ABI System, USA) following the amplification protocol specified by Sobhy et al. [36]. The β-actin gene was used as a reference housekeeping gene to determine the relative expression of the studied genes [37].

### Statistical analysis

The obtained results were reported as the mean ± the standard deviation of at least three independent replicates. Data means were separated by conducting a one-way analysis of variance (ANOVA) utilizing the CoStat software (v 6.311, CoHort). The significance of the mean differences was assessed using Duncan’s test at a significance level of 0.05. Unless otherwise specified, a significance level of *P* < 0.05 was considered significant. The Pareson correlation matrix heatmap was created using GraphPad Prism software (v 8.3.0).

## Results

### Phytochemical composition of the *Azolla* aqueous extract (AAE)

The phytochemical analysis using gas chromatography-mass spectrometry (GC-MS) analysis of the *Azolla* aqueous extract (AAE) identified a total of 50 distinct compounds, as depicted in Table 2 and Supplementary Fig. 1. The composition of the extract was characterized by a diverse assemblage of compounds, with most of them being present in miniature concentrations. Notably, the versatile components that were found in relatively higher abundance within the AAE included γ-aminobutyric acid (9.35%), 1,2-benzenedicarboxylic acid, dicyclohexyl ester (7.22%), n-hexadecanoic acid (5.27%), diisooctyl phthalate (2.86%), dibutyl phthalate (3.48%), 9-octadecene, (E)- (2.46%), 3-octadecyne (2.24%), butylated hydroxytoluene (2.09%), diphenylamine (1.43%), and tetratetracontane (1.27%). Additionally, the AAE exhibited the presence of various other phytochemicals, albeit in intermediate quantities that were relatively lower compared to the aforementioned compounds.

**Table 2:** The phytochemical constituents of *Azolla* aqueous extract as identified by GC-MS

Peak no	RT (min)	Area %	Compound name	Molecular formula
1	6.404	0.393	Furan, 2,4-dimethyl-	C_6_H_8_O
2	7.879	1.208	Benzaldehyde, 3-methyl-	C_8_H_8_O
3	8.279	0.710	5-Formyl-6-methyl-4,5-dihydropyran	C_7_H_10_O_2_
5	8.794	0.382	2-Butoxyethyl acetate	C_8_H_16_O_3_
6	9.385	9.354	γ-aminobutyric acid	C_4_H_9_NO_2_
8	11.030	0.328	3-Pentanamine	C_5_H_13_N
9	11.415	0.654	Octanoic Acid	C_8_H_16_O_2_
11	13.606	0.884	Nonanoic acid	C_9_H_18_O_2_
12	17.268	2.093	Butylated Hydroxytoluene	C_15_H_24_O
13	17.328	0.272	2,5-Cyclohexadiene-1,4-dione, 2,6-bis(1,1- dimethylethyl)-	C_14_H_20_O_2_
14	19.259	1.239	3-Octadecene, (E)-	C_18_H_36_
15	19.699	1.431	Diphenylamine	C_12_H_11_N
17	21.810	2.463	9-Octadecene, (E)-	C_18_H_36_
18	21.990	0.370	Malonic acid, 3-methylbutyl octyl ester	C_16_H_30_O_4_
19	22.425	2.244	3-Octadecyne	C_18_H_36_
20	22.780	0.875	Citramalic acid	C_5_H_8_O_5_
22	23.645	0.255	Malonic acid, isobutyl nonyl ester	C_16_H_30_O_4_
23	23.855	0.277	Tridecanoic acid, methyl ester	C_14_H_28_O
24	24.361	3.482	Dibutyl phthalate	C_16_H_22_O_4_
25	24.431	5.271	n-Hexadecanoic acid	C_16_H_32_O_2_
26	24.961	0.349	Oxalic acid allyl octadecyl ester	C_13_H_22_O_4_
27	25.046	1.043	5-Octadecene, (E)-	C_18_H_36_
28	27.047	0.452	Phytol	C_20_H_40_O
29	27.507	0.986	4-Tridecene, (Z)-	C_14_H_28_
30	27.957	0.898	Myristic acid	C_14_H_28_O_2_
31	28.562	0.304	Octadecyl trifluoroacetate	C_20_H_37_F_3_O
32	33.889	7.221	1,2-Benzenedicarboxylic acid, dicyclohexyl ester	C_20_H_26_O_4_
33	34.040	0.449	Hexanoic acid, 2,7-dimethyloct-7-en-5-yn-4-yl ester	C_16_H_26_O_2_
34	34.420	2.863	Diisooctyl phthalate	C_24_H_38_O_4_
35	34.540	0.881	2,4,6-Cycloheptatrien-1-one, 3,5-bis-trimethylsilyl-	C_13_H_22_OSi_2_
38	36.601	0.264	Sinapic acid	C_11_H_12_O_5_
39	37.666	0.396	1,2-Benzisothiazol-3-amine tbdms	C_13_H_20_N_2_SSi
40	37.751	0.266	Benzoic acid, 3,5-bis(1,1-dimethylethyl)-4-hydroxy-	C_15_H_22_O_3_
41	37.786	0.487	2-t-Butyl-5-(dimethoxy-phosphoryl)-3-methyl-4- oxoimidazolidine-1-carboxylic acid, t-butyl ester	C_15_H_29_N_2_O_6_P
42	37.901	1.273	Tetratetracontane	C_44_H_90_
43	37.996	0.408	Cyclotrisiloxane, hexamethyl-	C_6_H_18_O_3_Si
44	38.096	0.528	1,2,4-Benzenetricarboxylic acid, 1,2-dimethyl ester	C_11_H_10_O_6_
45	38.156	0.470	Cyclotetrasiloxane, octamethyl-	C_8_H_24_O_4_Si_4_
46	38.221	0.522	5-Methyl-2-trimethylsilyloxy-acetophenone	C_12_H_18_O_2_Si
47	38.271	0.265	Purine-2,6-dione, 8-(3-ethoxypropylamino)-1,3- dimethyl-3,9-dihydro-	C_12_H_19_N_5_O_3_
48	38.346	0.470	Cyclobarbital	C_12_H_16_N_2_O_3_
49	38.416	0.282	2’,6’-Dihydroxyacetophenone, bis(trimethylsilyl) ether	C_14_H_24_O_3_Si_2_
50	38.486	0.398	trans-4-(2-(5-Nitro-2-furyl)vinyl)-2-quinolinamine	C_15_H_11_N_3_O_3_

RT = Retention time

### Mineral content

The concentration of mineral ions in wheat seedlings (*Triticum aestivum* L. cv. Sakha 96) treated with salt stress (250 mM NaCl), AAE (foliar and priming treatments), either individually or combined with the salt treatment, is shown in Table 3. The results indicated that administration of 250 mM NaCl significantly lowered the nitrogen (N), phosphorus (P), calcium (Ca), and potassium (K) levels in wheat seedlings. In contrast, the seedlings exposed to salt stress exhibited a significant rise in the levels of magnesium (Mg) and sodium (Na), compared to their levels in the control seedlings.

**Table 3:** The effect of priming and foliar spraying with *Azolla* aqueous extract on the ionic content of salt-stressed wheat seedlings. Different letters within the same column imply significant differences at 5% level as determined by Duncan’s test

Treatments	Ionic content (mg g^− 1^ DM)							Ratio
	*N*	*P*	Ca	Mg	K	Na		K/Na
**Control**	0.110 ± 0.01^c^	0.037 ± 0.00^b^	0.53 ± 0.04^a^	0.27 ± 0.02^b^	4.37 ± 0.08^a^	0.39 ± 0.01^c^		11.23 ± 0.30^a^
**NaCl**	0.036 ± 0.00^e^	0.030 ± 0.00^c^	0.41 ± 0.03^c^	0.38 ± 0.02^a^	2.22 ± 0.08^e^	0.73 ± 0.02^a^		3.05 ± 0.03^e^
**AAE spray**	0.175 ± 0.00^a^	0.047 ± 0.00^a^	0.53 ± 0.03^a^	0.21 ± 0.03^c^	3.31 ± 0.05^b^	0.30 ± 0.00^d^		11.06 ± 0.13^a^
**NaCl + AAE spray**	0.051 ± 0.01^d^	0.039 ± 0.00^b^	0.44 ± 0.02^bc^	0.20 ± 0.02^c^	2.61 ± 0.04^d^	0.50 ± 0.02^b^		5.19 ± 0.23^d^
**AAE priming**	0.152 ± 0.00^b^	0.044 ± 0.00^ab^	0.51 ± 0.03^a^	0.15 ± 0.01^d^	3.06 ± 0.04^c^	0.31 ± 0.02^d^		9.89 ± 0.54^b^
**NaCl + AAE priming**	0.107 ± 0.01^c^	0.039 ± 0.01^b^	0.49 ± 0.01^ab^	0.22 ± 0.01^c^	3.11 ± 0.07^c^	0.49 ± 0.02^b^		6.39 ± 0.32^c^
	**Source of variance**							
**F**	268.38	6.54	8.99	56.72	449.69	295.28		374.15
**P**	***	**	***	***	***	***		***
**LSD at 5%**	0.010	0.007	0.050	0.033	0.106	0.028		0.543

*** = highly significant at 5% level; ** = significant at 5% level

Incredibly, the application of a single AAE treatment, whether through priming or spray, led to an increase in N and P levels. However, it caused a decrease in magnesium Mg, K, and Na contents to levels lower than that of the control, but the Ca level was marginally impacted. The coupled interaction of AAE with salt-stressed wheat seedlings significantly exacerbated the negative impact caused by NaCl on the levels of N, P, Ca, and K, with their levels, in some circumstances, close to those of the control. Nevertheless, these interactions resulted in a greater decrease in the level of Mg relative to both the control and the salt-stressed wheat seedlings. However, these treatments demonstrated a significant reduction in the concentration of Na ions in stressed seedlings, resulting in a mitigating effect on the accumulation of Na ions.

The K/Na ratio exhibited a significant decrease in response to salt stress. Nevertheless, the decrease in responsiveness to AAE single treatments was either ineffective or somewhat effective compared to the control ratio, but these combined treatments reflected a partial restoration of the K/Na ratio to levels higher than that observed in seedlings subjected alone to salt stress. Overall, the application of AAE resulted in enriching wheat seedlings with mineral ions, except K, which improved by spraying AAE as compared to the priming treatment.

### Antioxidant status

**Fig. 1:**
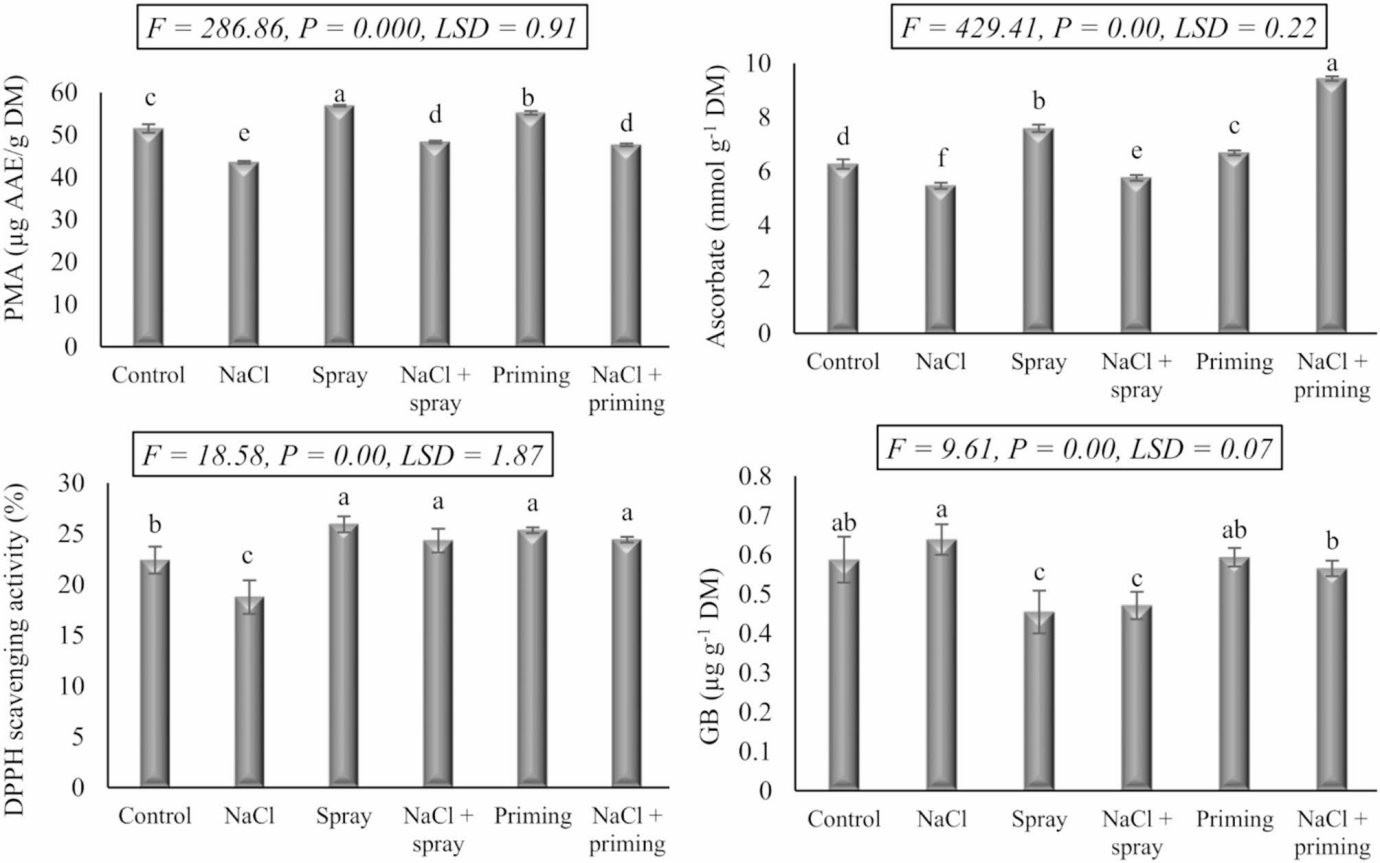
The effect of priming and foliar spraying with *Azolla* extract on the antioxidant capacity of salt-stressed wheat seedlings. Different letters imply significant differences at 5% level as determined by Duncan’s test

The data depicted in Fig. 1 highlights the impact of salt stress (250 mM NaCl) and the application of AAE (both via foliar and priming methods) on the antioxidant activity (DPPH scavenging and total antioxidant capacity) and the level of the anti-stress compounds (ascorbic acid and glycine betaine) in wheat seedlings. Highly significant variations (*P* < 0.01) in the assessed parameters were observed across the investigated treatments. Exposure of wheat seedlings to NaCl-induced salinity led to significant reductions in the total antioxidant capacity (TAC), DPPH scavenging activity, and ascorbic acid (15.46, 16.28, and 24.66%, respectively) compared to control levels. In contrast, salt stress triggered a rise in glycine betaine (GB) level by 8.65% above those observed in control seedlings.

When applied individually, both foliar and priming AAE treatments resulted in a relative augmentation of TAC, DPPH scavenging, and ascorbic acid levels in wheat seedlings while diminishing the accumulation of GB compared to the control group. Nonetheless, the combined administration of salt stress and AAE exhibited a positive influence in mitigating the detrimental effects of salt stress on wheat seedlings. These combined treatments assisted in the restoration of diminished TAC, DPPH scavenging activity, and ascorbic acid level, occasionally surpassing control levels, particularly when salt-stressed wheat seedlings were primed with AAE. Conversely, regarding the level of GB, the application of AAE led to reduced levels below that of the control. Notably, the application of AAE as foliar spraying on stressed wheat seedlings effectively decreased GB content compared to the analogous priming treatment.

### Secondary metabolites

**Fig. 2:**
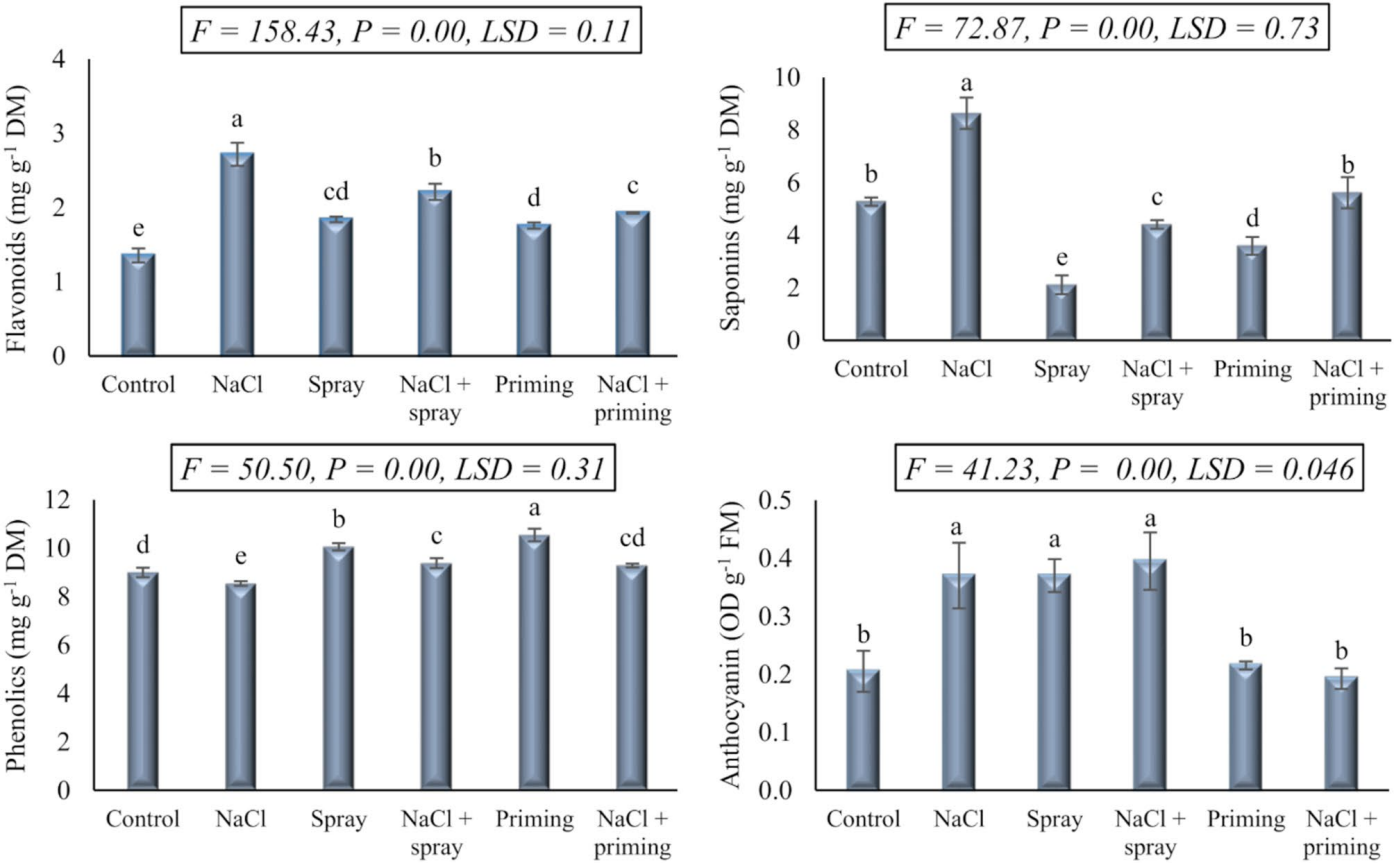
The effect of priming and foliar spraying with *Azolla* extract on the secondary active metabolites of salt-stressed wheat seedlings. Different letters imply significant differences at 5% level as determined by Duncan’s test

The variations in the concentrations of the investigated secondary metabolites, viz., flavonoids, phenols, saponins, and anthocyanins following the application of the investigated treatments on wheat seedlings, including salt stress (250 mM NaCl), AAE foliar spray and priming, as well as their combined administration, are depicted in Fig. 2. Highly significant variations (*P* < 0.01) in the accumulation of these secondary metabolites in wheat seedlings due to the experimental treatments were reported. The application of NaCl led to a significant increase in flavonoids (100.81%), saponins (63.76%), and anthocyanins (80.49%), albeit marginally decreasing phenol levels (5.00%) compared to control seedlings.

Individually administered AAE treatments exhibited a significant enhancement in flavonoids and phenols content, surpassing both control and salt-stressed treatments. While the AAE spray treatments significantly reduced saponin levels, while enhanced anthocyanin content in comparison to control values. Conversely, the AAE priming treatment decreased saponin content without a discernible impact on anthocyanin levels, as comparable to the control. The combined interactions of AAE with salt stress treatment assisted the restoration of flavonoids and saponins levels to approximate those of the control, countering the decline induced by salt treatment. In contrast, the combined application of NaCl and AAE treatments resulted in a notable increase in phenol levels, surpassing those of both the control and sole NaCl treatments. Intriguingly, the anthocyanin levels in seedlings treated with NaCl and AAE as a foliar spray remained unaltered from those in NaCl-stressed seedlings, maintaining a level analogous to that of the control in the case of the combined interaction between NaCl and AAE treatment.

### qRT-PCR analysis of the investigated genes

**Fig. 3:**
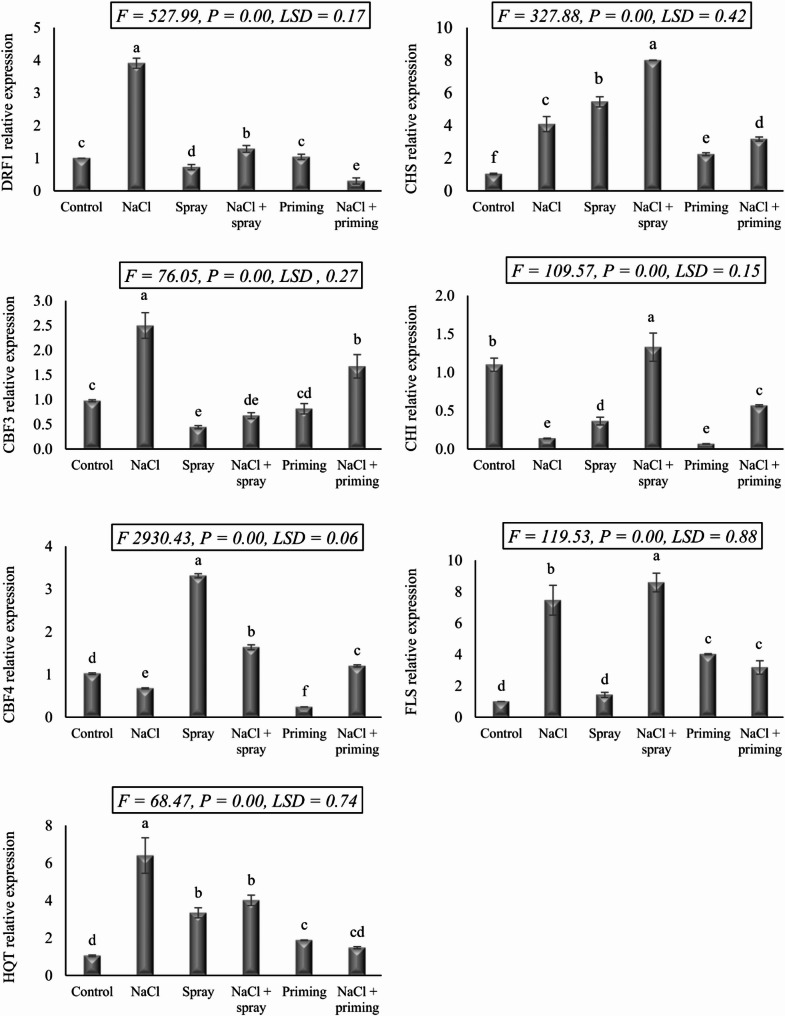
The effect of priming and foliar spraying with *Azolla* extract on the gene expression of salt-stressed wheat seedlings. Different letters imply significant differences at 5% level as determined by Duncan’s test

The results detailed in Fig. 3 investigate the intricate molecular responses exhibited by wheat seedlings when subjected to salt treatment, AAE priming, and foliar application, along with their interactive interactions. In the present investigation, we examined the transcriptional activities of seven pivotal genes in wheat seedlings, namely dehydration responsive factor 1 (DRF1), C-repeat binding factor 3 (CBF3), C-repeat binding factor 4 (CBF4), hydroxycinnamoyl-CoA quinate hydroxycinnamoyl transferase (HQT), chalcone synthase (CHS), chalcone isomerase (CHI), and flavonol synthase (FLS). The expression levels of these genes were high significantly (*P* < 0.01) influenced by the implemented experimental interventions.

Under salt stress, our findings unveiled a substantial upregulation in the expression of DRF1, CBF3, HQT, CHS, and FLS by 2.91, 1.52, 5.35, 3.06, and 6.45-fold, respectively, in response to 250 mM NaCl. Conversely, the expression profiles of CBF4 and CHI exhibited downregulation of 0.34 and 0.96-fold compared to the salt treatment. The application of AAE as a foliar spray on wheat plants elicited a notable upregulation in the expression levels of CBF4 (2.29-fold), HQT (2.29-fold), CHS (4.43-fold), and FLS (0.42-fold), while concurrently downregulated the expression of DRF1, CBF3, and CHI by 0.27, 0.53, and 0.73-fold, respectively. Priming with AAE triggered a decline in the expression of CBF3, CBF4, and CHI by 0.16, 0.78, and 1.03-fold, respectively, contrasted with an increase in the expression of HQT, CHS, and FLS by 0.83, 1.22, and 3.10-fold, respectively, with no obvious variation observed in DRF1 gene expression.

The interplay between salt stress and AAE treatments prompted various variations in the expression profiles of the studied genes. Remarkably, the interactive treatments of priming or foliar application of AAE with NaCl treatment resulted in a reduction in the expression of DRF1, CBF3, CBF4, and HQT genes below the levels observed in salt-stressed plants, particularly pronounced when AAE was utilized as a priming treatment in combination with salt stress. Conversely, the combined treatments of AAE and salt stress triggered an upregulated expression of the CHI gene, surpassing that induced solely by salt treatment, with foliar application yielding expression levels surpassing those of the control. Nevertheless, the effects of both AAE treatments on CHS and FLS genes’ expressions were disparate, with foliar application stimulating their upregulation and priming application inducing downregulation compared to their levels in salt-stressed wheat seedlings. In some instances, such as CHS, CHI, and FLS, the expression levels resulting from the combined treatment of AAE spray and salinity surpassed those of the control and salt stress treatments.

### Analysis of the heatmap of the evaluated attributes

**Fig. 4:**
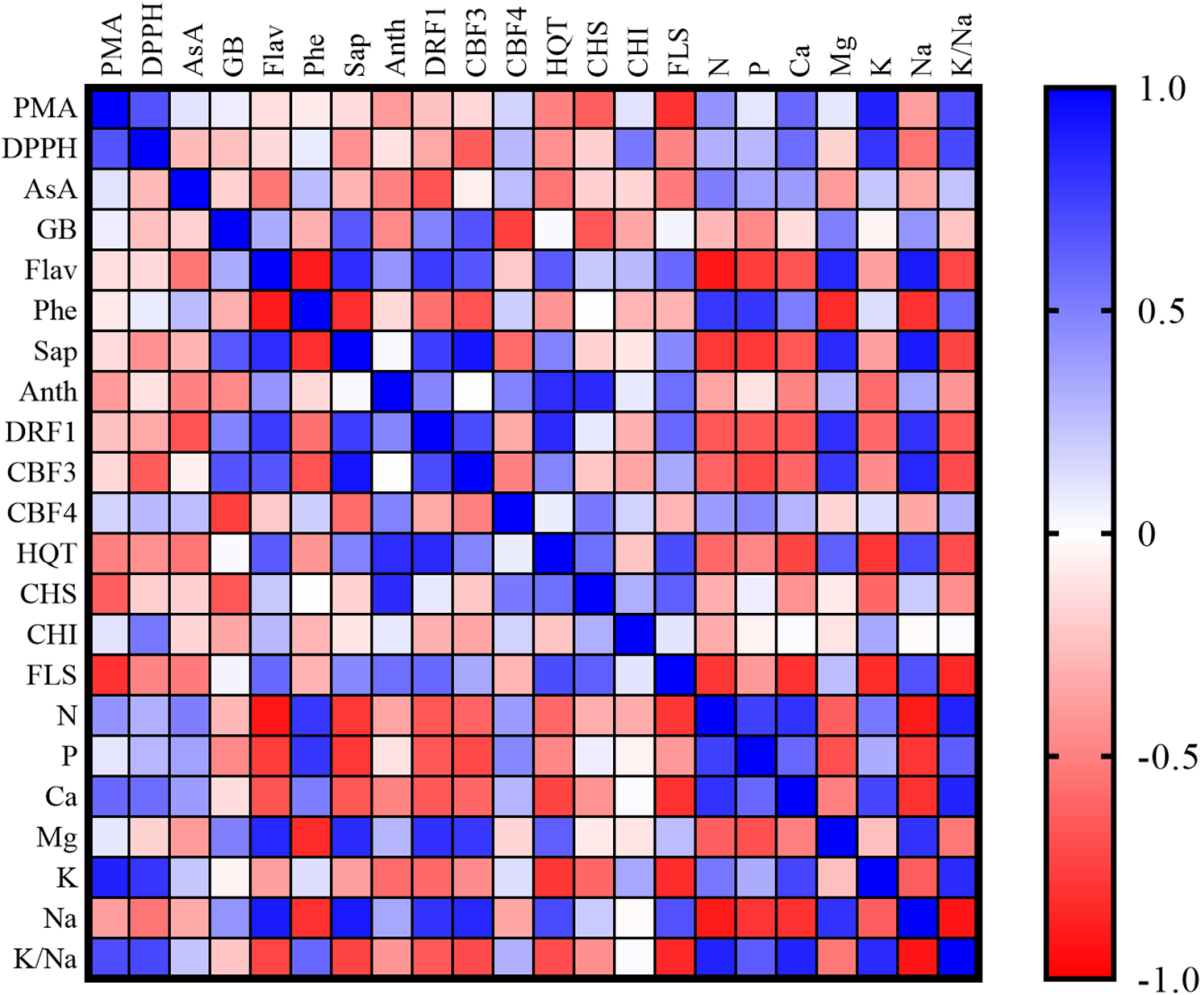
The heatmap analysis, generated from the correlation matrix of the assessed traits in wheat seedlings subjected to 250 mM NaCl, AAE priming, AAE foliar application, and their various combined treatments elucidating how these treatments interact to influence the plant’s stress responses based on gene expression patterns and physiological adaptations that enhance wheat resilience to salt stress. The abbreviations used are as follows: PMA for phosphomolybdate assay, AsA for ascorbate, GB for glycine betaine, Flav for flavonoids, Phe for phenols, Sap for saponins, and Anth for anthocyanins

The study assessed the interplay among the examined traits of wheat seedlings under salt treatment, AAE priming, and foliar application, including their combined effects, using heatmap visualization (Fig. 4). The findings indicated significant positive correlations among various characteristics. Noteworthy positive associations included the correlations of PMA with DPPH, Ca, K, and K/Na. DPPH exhibited significant positive correlations with K and K/Na, while GB displayed significant positive relationships with CBF3 and saponins. Flavonoids were positively correlated with DRF1, CBF3, Na, Mg, and saponins. Phenols also displayed significant positive correlations with N, P, and K/Na, whereas saponins exhibited strong positive correlations with DRF1, CBF3, Mg, and Na. Furthermore, the heatmap analysis unveiled additional significant positive correlations, such as the association between anthocyanins and HQT as well as CHS, DRF1 with CBF3, HQT, Mg, and Na, CBF3 with Mg, and Na, and HQT with FLS, Mg, and Na.

Conversely, the analysis of the correlation matrix-based heatmap revealed numerous significant negative correlations. For instance, the relationship between flavonoids and phenols, N, P, Ca, and K/Na exhibited significant negative associations. Additionally, the correlation of phenols with saponins, CBF3, Mg, and Na, and of saponins with N, P, Ca, and K/Na displayed significant negative correlations. Similarly, the correlation of DRF1 with N, P, Ca, K/Na, and of P with Mg, Na, and K/Na were characterized by significant negative correlations.

## Discussion

Within its ecological niche, the plant, as a sessile organism, encounters formidable challenges posed by its surrounding environment, notably in the context of a changing climate. To route these obstacles, plants have intricately improved specific adaptive mechanisms. Nevertheless, the incongruity between these evolved strategies and the rapidly changing environment detrimentally impacts the growth and yield rates of plants, particularly within crop species. In response to this predicament, a multitude of experiments have been orchestrated by plant scientists and agronomists aimed at augmenting plant resilience and acclimatization to environmental challenges. These attempts involve a range of approaches such as synthetic growth stimulants, natural extracts, symbiotic associations, different soil amendments, and nanoparticles. Among these avenues, natural extracts emerge as an outstanding candidate, characterized by their eco-friendly, safe, and cost-effective characteristics, thus presenting a reasonable solution for the application.

### Phytochemical analysis of AAE

The phytochemical analysis of *Azolla aqueous extract* (AAE) using GC-MS technique revealed a diverse array of phytocompounds. γ-aminobutyric acid, 1,2-benzenedicarboxylic acid, dicyclohexyl ester, and n-hexadecanoic acid were notable components, along with several others present in minor concentrations within the AAE. The utilization of γ-aminobutyric acid (γ-ABA) demonstrated efficacy in preserving growth and yield responses in plants experiencing salt stress, effectively counteracting the inhibitory effects on growth, physiology, and yield induced by salt stress [38]. Additionally, γ-ABA exhibited the capacity to ameliorate salt-induced impairments in apple seedlings by stimulating chlorophyll synthesis, augmenting photosynthetic efficiency, suppressing ROS accumulation, maintaining ion equilibrium, and upregulating genes associated with Na^+^ and K^+^ transport under salt stress conditions [39]. Consequently, the application of γ-ABA may emerge as a fundamental approach in ameliorating the deleterious consequences of salt stress and fostering the development of resilient genotypes. Benzenedicarboxylic acid (BDA), a phytochemical constituent in AAE, has been extensively studied for its role in directing plant physiological adjustments toward a redefined equilibrium. The protective effects of BDA include the stimulation of antioxidant defense mechanisms, enhancement of enzymatic activities, and enhancement of the nutritional profile of stressed plants [40]. Through mechanisms such as the suppression of ROS production, fortification of antioxidative pathways, and regulation of osmoregulatory compounds biosynthesis, the external application of BDA bolstered plant resilience to combined abiotic stressors [41].

### Ionic content

In the current investigation, exposure of wheat at the seedling stage to 250 mM NaCl salt stress notably reduced nitrogen (N), phosphorus (P), calcium (Ca), and potassium (K) levels while increasing magnesium (Mg) and sodium (Na) levels. Generally, salt stress has been reported to reduce the uptake of essential ions like N, P, K, and Ca [42, 43]. The elevated Na level has been established to induce nitrogen deficiency across various plant species. Salinity instigates a progressive reduction in N uptake and metabolic processes. The stress induced by NaCl disrupts the enzymatic functions crucial for nitrogen metabolism, thereby impeding the assimilation and utilization of nitrogen in plants [44].

Furthermore, increased soil salinity leads to the sequestration of P in plants due to its precipitation with other cations, thereby instigating soil-induced phosphorus deficiency in plants [45]. The high Na level within the root zone obstructs the uptake and translocation of Ca, with Na ions potentially outcompeting Ca ions for binding sites on membranes [46]. Also, Na ions exhibit notable competitive inhibition against K ions owing to their similar ionic radius and hydration energy. Consequently, the substantial influx of external Na ions impedes the influx of potassium ions, resulting in K deficiency [47]. The increased Mg in wheat following salinity exposure is compatible with the findings of [48] in alfalfa. The rise in Mg level can be attributed to its role in activating enzymes necessary for stress response and adaptation, aiding in osmotic adjustment and maintaining cell turgor pressure, and boosting the plant’s antioxidant defense mechanisms, consequently mitigating the harmful impacts of salt stress.

Application of AAE, alone or in combination with salt stress, led to varied effects on mineral ion levels, with AAE mitigating Na accumulation and enhancing N and P levels, but exacerbating the decrease in Mg levels. The K/Na ratio decreased under salt stress, with combined AAE treatments showing potential for restoring this ratio to levels surpassing those of salt-stressed seedlings alone, enhancing wheat seedlings with mineral ions. *Azolla* is well-known for its significant mineral content, including Ca, N, P, K, Fe, Mn, Cu, and Zn [49]. The significant level of nutrients in AAE may assist in alleviating the ionic imbalance induced by salt in wheat plants. Furthermore, the enhancement of nutrient content in salt-affected wheat seedlings by AAE can be attributed to the ability of this extract to increase nutrient availability and uptake, even in stressful conditions, due to its potential to lower soil pH, enhance soil organic matter, and improve the physical, chemical, and biological characteristics of the soil [50].

### Antioxidant status

Exposure to NaCl-induced salinity reduced antioxidant status (TAC and DDPH) and ascorbic acid levels while increasing GB in wheat seedlings. The decreased TAC and DPPH activity in this study could be ascribed to the decreased level of phenolic compounds following salinity-stress exposure, and the excessive ROS production which exceeded the plant antioxidative potential. The diminished levels of DPPH and TAC indicate a malfunction in the ROS scavenging mechanism, resulting in an imbalance between generated antioxidants and ROS. Additionally, under higher NaCl concentrations, the enzymatic antioxidant system may exhibit heightened efficacy, potentially making the plant-dependent principally on enzymatic rather than non-enzymatic antioxidant pathways [51].

Salt stress can reduce the concentration of ascorbic acid in wheat plants due to excessive oxidative stress, which can disrupt the enzymatic pathways crucial for synthesizing ascorbic acid. Moreover, an ion imbalance induced by salt stress can impede the uptake and transportation of essential nutrients involved in ascorbic acid biosynthesis. Furthermore, the metabolic changes triggered by salt stress can divert resources towards stress response mechanisms, potentially diminishing ascorbic acid production in wheat plants under saline conditions. Moreover, the excessive accumulation of GB can potentially boost or stabilize the activity of antioxidant enzymes, reduce oxidative injury to biomembranes, and promote better ion homeostasis [52]. Moreover, it has been established that the accumulation of GB under salty conditions inhibits the generation of ROS and promotes the accumulation of osmoregulatory molecules such as glutathione and proline [53].

Foliar and priming treatments with AAE boosted TAC, DPPH activity, and ascorbic acid levels and decreased GB accumulation. Also, combined salt stress and AAE treatments effectively restored antioxidant potentials, with AAE foliar application showing better reduction of GB compared to priming treatment. The beneficial impact of AAE on the antioxidative capacity of wheat seedlings, involving the enhancing of DPPH, TAC, ascorbic acid levels, and the reduction of GB accumulation induced by salt stress, was explained by the extract’s ability to improve the metabolic efficacy, regulate antioxidant enzyme activities, and normalize the expression levels of stress-related genes in wheat seedlings [8]. Maswada et al. [54] demonstrated that AAE plays a crucial role in enhancing water status, osmotic adjustment, membrane stability, and reducing lipid peroxidation in stressed plants, resulting in improved oxidation status and decreased accumulation of osmoregulatory molecules like GB. Furthermore, the application of *Azolla* as a soil amendment increased soil essential nutrients, enhanced microbial activity, and improved soil properties such as water retention, porosity, and infiltration rate [55, 56]. This leads to reduced oxidative stress and obviates the need for additional osmoregulatory compounds, ultimately facilitating the restoration of oxidative potential in stressed wheat seedlings through metabolic pathway adjustments.

### Secondary metabolites

In the current investigation, the application of NaCl (250 mM) significantly increased flavonoids, saponins, and anthocyanins, while slightly decreasing phenols compared to control values. The alternation in the secondary metabolomics profile, encompassing polyphenolics, flavonoids, saponins, anthocyanins, and tannins, has been documented in some plant species subjected to salinity stress. Some of these secondary metabolites may exhibit hormone-like properties, fostering enhanced tolerance to salinity stress. Under salt stress circumstances, there has been a documented increase in the expression of genes such as PAL, CHS, and FLS, that are involved in the production of flavonoids. This increase in gene expression is correlated with the synthesis of anthocyanins, which enhances the plant’s ability to withstand stress [57].

Additionally, it is postulated that ABA may trigger the accumulation of flavonoids by modulating the enzymatic activity of flavonoid metabolism in response to abiotic stress [58]. Anthocyanins have been identified as protective agents against oxidative stress induced by salinity [59]. At elevated salinity levels, the accrual of anthocyanins might positively impact the safeguarding of the photosynthetic apparatus [60]. Consequently, the induction of anthocyanin synthesis in response to salinity stress could be regulated by the activation of the phenylpropanoid pathway. Plants demonstrating heightened saponin production have been shown to exhibit enhanced growth under saline conditions [61]. The escalated activation of NADPH oxidase results in the excessive consumption of NADPH, crucial for squalene synthesis within the steroid synthesis pathway, which is pivotal for saponin biosynthesis [62]. The slight reduction in phenols observed in this investigation could be elucidated by the heightened activity of polyphenol oxidase, the increased utilization of phenols for lignin synthesis, and the increased production of flavonoids and anthocyanins in salt-stressed wheat seedlings.

The analysis of secondary metabolites showed that treating with AAE alone or in combination with salt stress caused a decrease in flavonoids and saponins, reaching levels similar to or lower than the control. Additionally, it increased the content of phenols above the levels of both the control and salt treatment. Spray treatments led to an increase in anthocyanin while priming treatments reduced its levels. The concentrations of secondary metabolites assessed in salt-stressed plants were reestablished following the administration of AAE. As per the data provided, either priming or foliar spray with AAE led to the restoration of the levels of these metabolites to values that, in most instances, closely approximated those of the control group. The decline in flavonoids and saponins noted in the current investigation subsequent to AAE application could be attributed to the high presence of antioxidants within this extract, which confers robust protection for developing wheat seedlings in both normal and saline environments. Consequently, there arises no necessity for their accumulation, prompting the plant to allocate its metabolic resources towards the production of other bioactive compounds crucial for optimal growth [8]. In an investigation on cotton plants subjected to salt stress conducted by Ibrahim [63], it was demonstrated that the application of AAE mitigated the deleterious impacts of salt stress on cotton plants by promoting the biochemical profile, particularly phenolics, and antioxidant capacities.

### qRT-PCR gene expression

The current findings showed significant gene expression changes under salt stress, with DRF1, CBF3, HQT, CHS, and FLS upregulated and CBF4 and CHI downregulated. In the context of salt stress, the heightened expression of DRF1 may arise from the osmotic stress induced by salinity, with DRF1 encoding pivotal transcription factors crucial in water-stress responses [64]. The ubiquity of DRF1 protein across various grass species, its regulation through alternative splicing in tissue-specific manners, and its involvement in stress gene modulation via an ABA-dependent pathway have been well documented [65]. In agreement with our results, CBF3 as a transcription factor linked to osmotic stress responses, exhibiting upregulation in response to salinity [66]. The reported mode of salt tolerance enabled by CBF3 involves direct interaction with the GALS1 promoter, suppressing its expression and fostering the accumulation of galactinol and raffinose, thus enhancing salt tolerance [67]. Moreover, CBF3 overexpression was reported to be associated with mitigating ROS generation in plants facing drought and salinity stress [68].

HQT, an enzyme controlling plant secondary metabolism through phenylpropanoid pathway regulation, is vital for growth and development. Overexpression of HQT can lead to elevated chlorogenic acid levels, enhancing antioxidant capacity and potentially aiding in maintaining membrane integrity during salt stress [69]. Additionally, CHS is a pivotal enzyme in flavonoid biosynthesis, and responds to various environmental cues, including salinity, through the phenylpropanoid pathway [70]. Upregulation of CHS is linked to increased flavonoid levels, known for their role in combating oxidative stress induced by salinity [71]. Alternation in CHS gene expression can influence the accumulation of metabolites such as anthocyanins, isoflavones, flavones, and flavonols [72]. FLS, crucial for flavonol synthesis, plays a significant role in salt stress responses by promoting ROS elimination through flavonol accumulation under salt stress conditions [73]. Additionally, FLS is involved in auxin transport mechanisms and environmental stress protection [74].

CBF4, a transcription factor induced by various abiotic stresses, enhances salt tolerance and upregulates downstream genes upon overexpression [75]. The response of wheat to salt stress may not profoundly rely on CBF4 expression levels, pointing towards the potential involvement of other genes and transcription factors in triggering antioxidative mechanisms against salinity-induced damage [76]. CHI, a key enzyme in the phenylpropanoid metabolic pathway, along with CHS, plays a crucial role in mitigating oxidative stress induced by salinity through increased flavonoid accumulation [77]. The decreased expression of CHI in wheat suggests a dependence on CHS, rather than CHI, for flavonoid accumulation under salt stress conditions [78].

The findings of the present investigation revealed that the application of AAE, whether as a priming agent or foliar spray, led to an increased expression of HQT, CHS, and FLS genes. This enhancement signified a bolstering of secondary metabolism pathways, notably the phenylpropanoid pathway mediated by HQT, which plays a pivotal role in the biosynthesis of flavonoids facilitated by CHS and FLS. The heightened expression of genes responsible for flavonoid biosynthesis was documented to counteract the cytotoxic impacts of ROS by effectively scavenging these ROS, thereby mitigating oxidative stress [79]. Furthermore, the escalated expression of PAL, CHS, FLS, and other genes downstream of flavonoids was linked to the enhancement of anthocyanin biosynthesis in response to salt stress, consequently fortifying the plant’s resilience to stress conditions [57]. These findings support the idea that AAE boosts the antioxidant defense mechanisms within wheat plants, in both normal and stressful conditions. The diminished expression of certain stress-responsive genes such as CBF3 and CHI after AAE treatment was ascribed to the abundant presence of antioxidant compounds in AAE, which empower plants to neutralize ROS, thereby redirecting their metabolic pathways towards the production of essential molecules crucial for growth [8].

The current study demonstrated that when AAE combined with salt stress, the equilibrium in the investigated genes was attained to reach values more or less than the control expression. This interaction reduced the expression of DRF1, CBF3, CBF4, and HQT genes while increasing CHI gene expression. However, the effect of AAE treatments on CHS and FLS gene expressions varied, with foliar application boosting their expression, while priming treatment downregulated their expression. This may be attributed to the extract’s nutrient, antioxidant, and biostimulant capabilities in response to imposed salt stress [54]. The diminished expression of DRF1, CBF3, CBF4, and HQT genes may be linked to the shielding influence of AAE against salinity-induced dehydration, osmotic stress, and the biosynthesis of secondary metabolites in wheat seedlings. This association could be attributed to the osmomodulatory and anti-stress properties of the extract, such as γ-aminobutyric acid and benzenedicarboxylic acid, which defend the plant against the detrimental consequences prompted by salt stress. Al-Huqail et al. [8] postulated that the repression of gene expression in salt-exposed wheat seedlings by AAE offers a strong indication that the extract possesses substantial antioxidant properties. This suggests that the extract’s natural antioxidant potentials render additional innate immunity mechanisms unnecessary when confronting salt-induced ion toxicity and oxidative stress. Consequently, AAE represents a viable approach to alleviate the detrimental impacts of the ionic and osmotic imbalances caused by salt stress in wheat seedlings. The modification in gene expressions associated with stress responses and production of secondary metabolites induced by AAE could signify the molecular resistance of wheat seedlings to salt stress [80, 81]. So, these findings underscore AAE’s potential as a sustainable solution for mitigating salt stress in wheat, offering significant implications for enhancing crop resilience and productivity in large-scale agricultural practices.

## Conclusion

The study demonstrated the potential of Azolla aqueous extract (AAE) in mitigating the adverse effects of salt stress on wheat seedlings. AAE treatments positively influenced the phytochemical composition, mineral content, antioxidant status, secondary metabolites, and gene expression profiles in response to salt stress. Notably, AAE application led to enriched mineral ion levels, enhanced antioxidant activity, and modulation of secondary metabolites. The interactive effects of AAE with salt stress showed complex variations in gene expression, with some genes being upregulated while others were downregulated compared to salt stress. These findings underscore the multifaceted benefits of AAE in enhancing plant resilience to salinity stress. Further research could delve into the underlying molecular mechanisms driving these responses and optimize AAE application strategies for improved crop stress tolerance. Overall, this study contributes valuable insights into utilizing AAE as a potential bioresource for enhancing plant stress resilience and agricultural sustainability in saline environments.

## Electronic supplementary material

Below is the link to the electronic supplementary material.

Supplementary Material 1

## Data Availability

The datasets used and/or analysed during the current study are available from the corresponding author on reasonable request.
