# Supplementary figures and images for: The interplay of salt stress and Azolla aqueous extract on ionic balance, secondary metabolism, and gene expression in wheat seedlings
Source: BMC Plant Biol. 2025 May 23;25:688. doi: 10.1186/s12870-025-06688-3 (PMC12101025; doi:10.1186/s12870-025-06688-3)

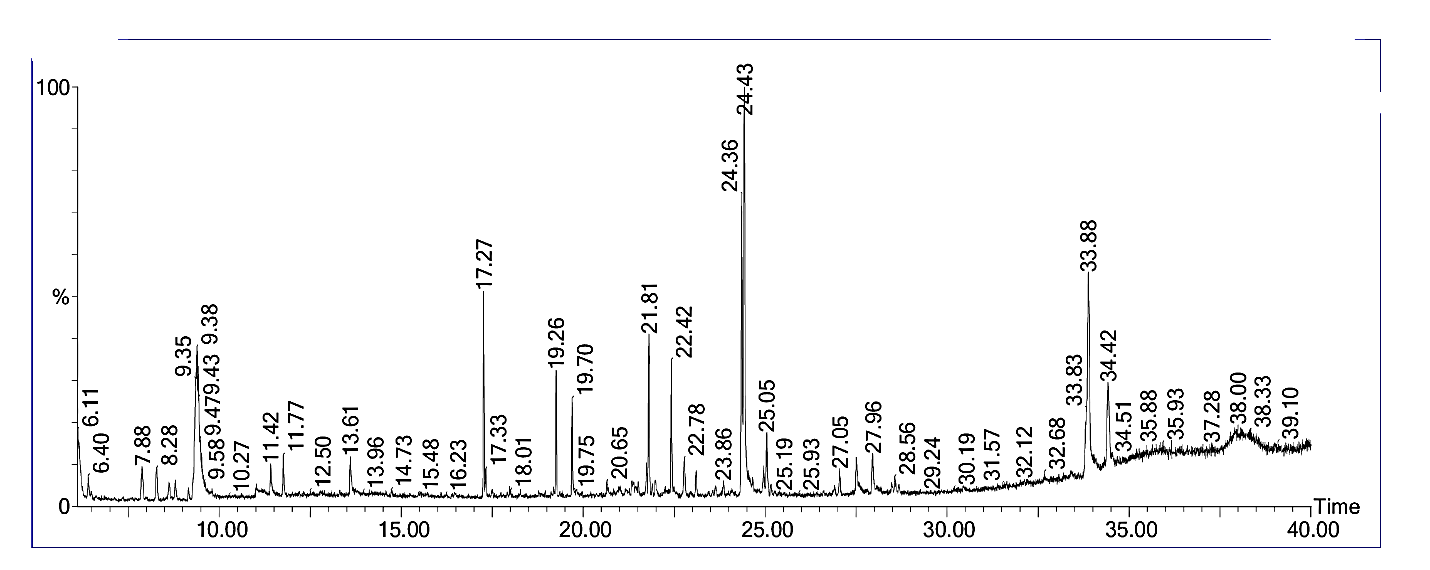

Supplement: Supplementary file 1 — Supplementary Material 1 [file 12870_2025_6688_MOESM1_ESM.docx]
